# Customizable 3D Printed ‘Plug and Play’ Millifluidic Devices for Programmable Fluidics

**DOI:** 10.1371/journal.pone.0141640

**Published:** 2015-11-11

**Authors:** Soichiro Tsuda, Hussain Jaffery, David Doran, Mohammad Hezwani, Phillip J. Robbins, Mari Yoshida, Leroy Cronin

**Affiliations:** 1 WestCHEM, School of Chemistry, University of Glasgow, Glasgow, United Kingdom; 2 Institute of Molecular Cell and Systems Biology, University of Glasgow, Glasgow, United Kingdom; University of Illinois at Chicago, UNITED STATES

## Abstract

Three dimensional (3D) printing is actively sought after in recent years as a promising novel technology to construct complex objects, which scope spans from nano- to over millimeter scale. Previously we utilized Fused deposition modeling (FDM)-based 3D printer to construct complex 3D chemical fluidic systems, and here we demonstrate the construction of 3D milli-fluidic structures for programmable liquid handling and control of biological samples. Basic fluidic operation devices, such as water-in-oil (W/O) droplet generators for producing compartmentalized mono-disperse droplets, sensor-integrated chamber for online monitoring of cellular growth, are presented. In addition, chemical surface treatment techniques are used to construct valve-based flow selector for liquid flow control and inter-connectable modular devices for networking fluidic parts. As such this work paves the way for complex operations, such as mixing, flow control, and monitoring of reaction / cell culture progress can be carried out by constructing both passive and active components in 3D printed structures, which designs can be shared online so that anyone with 3D printers can reproduce them by themselves.

## Introduction

Three dimensional (3D) printing has long been developed in manufacturing industries, termed as Additive manufacturing (AM) [1]. While AM has been focusing mainly on a larger-scale industrial applications, 3D printing is more actively sought after in recent years as a promising novel technology to construct complex objects in nano- to millimeter scale. The scope of this technology is diverse: Nanotechnology [2], chemistry [3], biomedical engineering [4], electronics [5], and microfluidics [6,7]. A variety of 3D printing methods has been developed to construct complex 3D structures in the past: Stereolithography (SLA), Fused deposition modelling (FDM), Selective laser sintering (SLS), PolyJet, and so on. Whilst a higher resolution and hence smaller feature size can be achieved with SLA and SLS, fluidic devices constructed by these methods require the removal of uncured materials to use the internal structures as fluidic channels. This becomes difficult especially when the structures are complex, and thus previous applications using these methods were often limited to the construction of 2D structures. On the other hand, FDM allows a straightforward means to fabricate complex 3D fluidic channel structures without the requirement of subsequent cleaning post printing. In this respect we have recently demonstrated the design and production of 3D printed reactionware using the relatively inert thermoplastic polypropylene (PP) as a highly configurable low-cost alternative to traditional glassware for organic and inorganic chemical reactions [8–13].

Herein, we demonstrate that 3D printed reactionware can be utilized for biological applications with fluidic control. Microfluidics, or Lab-On-a-Chip (LOC) technologies have been increasingly applied to biological research in the last decade. While microfluidics mostly focuses on a single cell level at the micrometer scale, we here focuses on a cell population level using commercial and relatively inexpensive 3D printer with sub-millimeter to resolution. Conventionally, fluidic control at the sub-millimeter to millimeter scale were relying on off-the-shelf commercial fluidic devices (e.g., plastic tubes and fittings). As shown previously [8,11,13,14], 3D printing allows us to flexibly customize fluidic devices as needed and even the CAD designs of prints can be shared online so that anyone can download and reproduce by themselves. In recent years, 3D printing technologies have been more and more used for micro-/milli-fluidic systems, such as 3D fabrication of silicone elastomer [15], SLA-based 3D printed microfluidic systems [16], inkjet 3D printing of microfluidic modules [17]. These devices were mostly designed for biological applications [18]. It has also been demonstrated that external components, such as membranes [19] and electrodes [20], can be integrated with 3D printed devices.

When compared to the chemical systems, application of 3D printing technology to biological systems give us another advantage, which is the wider choices of printing materials: While complex chemical reactions often require solvents that are not compatible with commonly used 3D printable thermoplastics, such as polylactic acid (PLA) and Acrylonitrile butadiene styrene (ABS), the mild conditions required for biological manipulations present no such limitations. This means that there are more choices for the printing materials to construct 3D fluidic structures for biological manipulations, which can be tailored depending on application requirements, such as surface property (hydrophobicity), optical transparency, and thermal resistivity. We utilize the wider choices of printing materials to chemically modify the surfaces of 3D printed fluidic parts, because the printing materials are commonly used polymers and therefore amenable to various chemical treatments. We show that surfaces of 3D printed parts can be treated with oxygen plasma to bond with flexible silicone-based polymer and with dichloromethane (DCM) vapor to create soft surfaces for inter-connectable modular parts. In fact, this is another advantage of FDM-based 3D printers over SLA-based printers because printing materials for SLA-based printers are mostly proprietary and chemical compositions are unknown, while those for FDM-based printers are well described.

In the following sections, we show that (1) the construction of various 3D fluidic devices for liquid handling and control of biological cells and culture media are made possible using FDM-based 3D printing, and that (2) chemical surface modifications of the printed parts are possible to enhance the surface properties of 3D printed devices. FDM-based 3D printing has an advantage over other 3D printing methods that are used in the previous literature because it can directly create void channel structures using inexpensive polymers as printing materials. Using this method, we constructed a water-in-oil droplet generator, sensor-integrated cell growth chamber, valve-based flow selector, and interconnectable modular devices. The latter two devices were made possible by utilizing chemical surface modifications of 3D printed parts, which we demonstrate for the first time here. We envisaged that complex operations, such as mixing, flow control, and monitoring of reaction progress can be carried out by constructing both passive and active components in 3D printed structures. In the longer term, this could be also combined with external robotic devices to fully automate the whole experimental workflow including: preparation, execution and analysis of experiments. Towards this goal, we have extended the concept of 3D printed chemical fluidics to low-cost, rapid-manufactured millifluidic cell culture devices that can be networked in a scalable manner.

## Results and Discussions

### Droplet generator

Droplet generators have been widely employed to create a massive number of monodisperse droplets at the interface of two immiscible liquids in a short period of time. While single cells are encapsulated in microfluidic droplets (e.g., for directed evolution of proteins [22]), a population of cells are encapsulated in a droplet with millifluidic devices for, such as, high-throughput screening of antibiotics [23], and rapid drug assay of minimum inhibitory condition [24].

In order to robustly create droplets, the surface property of device material is crucial. To create two-phase water-in-oil (W/O) droplets, the device surface needs to be hydrophobic, otherwise it results in co-flowing of two streams or generation of unstable polydisperse droplets. We have investigated several commonly used thermoplastics for 3D printing: acrylonitrile butadiene styrene (ABS), polyethylene terephthalate (PET), polylactic acid (PLA), high-impact polystyrene (HIPS), and nylon (Taulman 618). We found that PLA is the best material for 3D printed droplet generator in terms of print reproducibility and reliable W/O droplets generation, as well as hydrophobicity.

**Fig 1:**
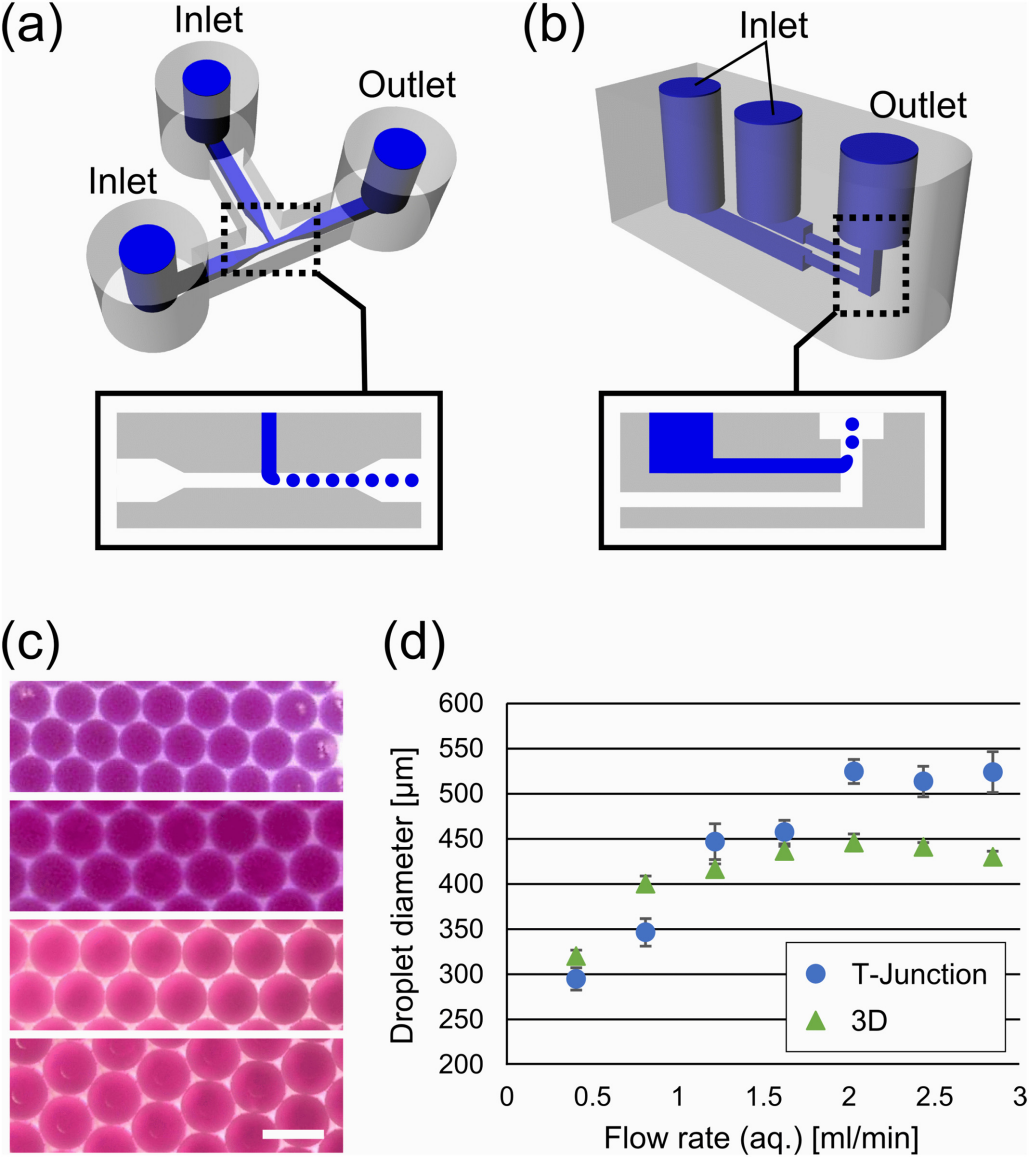
3D printed droplet generators. (A) CAD design of the T-junction device with one aqueous inputs, one oil input, and an output. Internal channel structures are illustrated in blue. Aqueous phase (indicated in blue in the inset) was introduced from the top inlet and oil phase (white) from the left. (B) The CAD design of 3D droplet generator. Aqueous phase (blue in the inset) was cut into small droplets by oil phase (white). (C) Microscopic pictures of generated droplets. Flow rates of aqueous phase are 0.4, 0.8, 1.6, and 2.4 ml/min (top to bot-tom). The top two images were from the 3D device, and the bottom ones were from the T-junction device. Scale bar: 250 μm. (D) Plot of droplet diameter as a function of the flow rate of aqueous phase. The flow rate of oil phase was fixed at 2.8 ml/min. Error bars were standard deviations.

Two droplet generators were designed to characterize droplets produced by the 3D printed reactionware devices. One of the designs is based on a conventional microfluidic T-junction device (Fig 1A). Another design utilizes the capability to construct 3D objects: water and oil channels are vertically aligned and droplets are generated at a vertical channel (Fig 1B). The dimensions of this device were 800 μm and 1.2 mm for the horizontal and vertical channels, respectively. The minimum resolution of 3D printed parts were determined to be ~400 μm on the CAD design (See S1 Fig for more details). Printing at the sub-millimeter resolution did not come out as designed, which can vary as much as 40%. However, the variation was consistent between prints (up to a C.V. of 6%. See S2 Fig).

By using a fluorinated oil, aqueous solution extruded from the top channel naturally forms droplets due to the lighter density than the oil. Fig 1C shows W/O droplets generated by the devices. Red dye was encapsulated in the droplets for visualization purpose. Both designs were found to be capable of producing quite monodisperse droplets. Fig 1D shows plots of the size of generated droplets against the flow rate for aqueous phase. Both devices generated droplets of similar diameters at lower flow rates, while they produced fixed size droplets at higher flow rates. We speculate that the fixed droplet diameter was due to the device geometry: As the droplet generator was constructed by rigid plastic, it created back pressure and hence the effective flow rate stayed constant. In contrast, flow rate was the dominant factor that determines the size in the lower flow rate region, and therefore the diameter of the droplets are proportional to the flow rate. It also should be noted that the monodispersity of droplets is quite high, as seen from the error bars in Fig 1D. The variance in droplet size was below 4% and 2% for T-junction and 3D droplet generators, respectively. However, generated droplets were highly polydisperse at an even lower flow rate (0.08 and 2.8 mL/min for aqueous and oil phases, respectively).

### Bacterial Culture Droplet System

**Fig 2:**
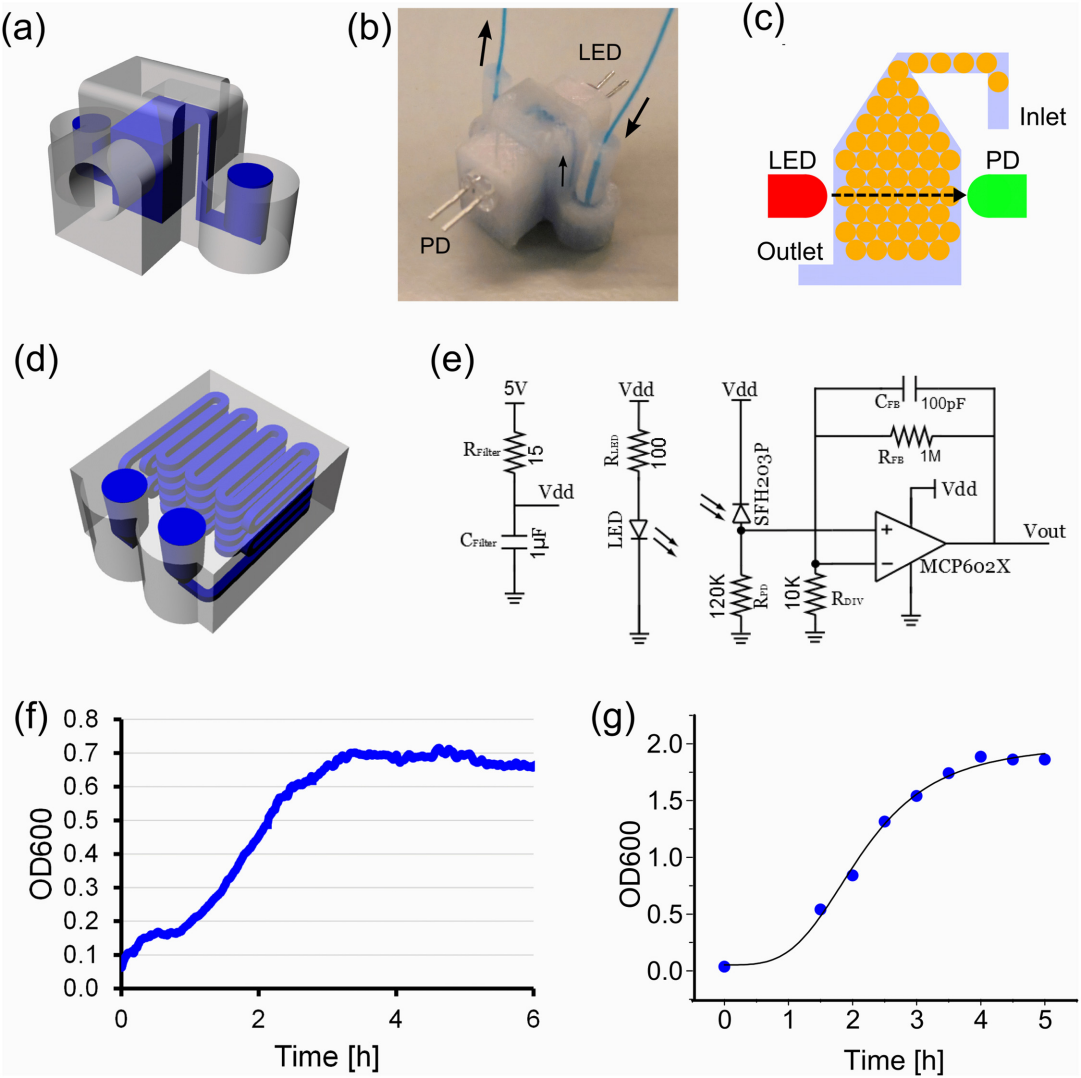
Droplet cell culture and monitoring devices. (A) CAD design of the droplet storage and growth measurement device. (B) A fabricated device with a LED and photodiode (PD) sensor attached. (C) Schematic diagram of droplet cell growth monitoring. Generated droplets are stored in the chamber as they float in the fluorinated oil. (D) CAD design of the long delay line droplet storage device. Dimensions of the device were 24.5×20.0×10.9 mm (W×D×H) (e) Op-amp circuit for PD signal amplification. Signals from V_out_ were read by the DAQ device. (F) A growth curve of *E*.*coli* cells in droplet stored in the 3D printed measurement device. (G) A growth curve of E.coli in droplets stored in the long delay-line device.

The millifluidic droplet generator was then employed to encapsulate bacterial cells and culture them inside droplets. In addition, we show that 3D printed devices enable the online monitoring of bacterial growth by integrating external light source and sensors in a 3D printed housing for optical density measurements. To achieve this *Escherichia coli* bacteria were cultured in a device comprised of a droplet generator and reservoir (Fig 2A–2C). Droplets encapsulating *E*.*coli* cells were generated in the T-junction device mentioned above at the flow rate of 0.8 and 2.8 ml/min (water and oil, respectively) and stored in the integrated reservoir. Two reservoir devices were designed: a 3D small chamber (Fig 2A–2C) and long delay line (Fig 2D). The former device was designed to collect droplets in the chamber by exploiting 3D structure. Here, Lower-density droplets introduced from the inlet floated in the higher-density oil phase and remained in the chamber. The chamber device was also equipped with a LED (λ_max_ = 600 nm) and photodiode sensor. The assembled device was kept in an incubator at 37°C and the growth of bacterial cells were collectively measured as the absorbance of LED light using the photodiode. Signals were amplified by an op-amp circuit (Fig 2E). The long delay-line device was used to simply store droplets in a 3D printed device. Several devices were loaded with droplets containing diluted cell culture and kept in the incubator. Droplets were collected every 30 min and OD at 600nm was measured using a Nanodrop 2000 spectrophotometer.

Fig 2F and 2g shows growth curves of bacterial cells in droplets cultured in the sensor-integrated 3D chamber and long delay-line devices, respectively. Although cells were cultured in similar condition (i.e. in-droplet culture), they resulted in different growth levels: OD_600_ ~ 0.7 in 3D chamber device, and OD_600_ ~ 1.8 in long delay-line device. The former result can be explained that a typical growth level of E.coli cells in the anaerobic condition would be below OD_600_ = 1 [25]. In fact, Jakiela et al observed that the growth of E.coli cells in W/O droplets saturates around at and OD_600_ ~ 700 [26]. Although the growth conditions are different (e.g., different E.coli strain and different growth medium), the result seems to be consistent with our results. Although the growth level started from non-zero values (OD_600_ ~ 0.065), this was due to the conversion from voltage readings to optical density (See S3 Fig for details). Hence, we assumed that there was no detectable growth in the first 1h in Fig 2F. On the other hand, the latter result indicates that the cells inside the long delay-line device were grown in or close to the aerobic condition, which is consistent with the aerobic growth in batch culture (S4 Fig). A possible explanation would be the porosity of the 3D printed parts: As the FDM-based 3D printer constructs a 3D structure by laying down molten plastic line by line, it inevitably leaves tiny gaps or pores between lines. They may have helped provide oxygen to the droplets, together with the relatively large surface-to-volume ratio of W/O droplets.

However, it should be noted that both growth curves showed logistic curves, which means the growth followed Monod’s bacterial growth model *N* = *N*_0_ *e* ^*kt*^ where *k* is a specific growth constant *k* = *k*_*max*_ *c*/(*K*_*s*_+*c*), *k*_*max*_ is the maximal growth constant, *c* is the concentration of the rate limiting nutrient, which is carbon source in the case of LB medium [27]. *K*_*s*_ is the saturation constant, which is equivalent to the substrate constant at *k* = *k*_*max*_/2 (i.e., the case when *c* = *K*_*s*_). By fitting the curves to exponential function, *k*_*max*_ was determined to be 1.1 and 2.18 h^-1^ for the 3D chamber and long delay-line devices, respectively.

### Flow control with 3D printed valves

**Fig 3:**
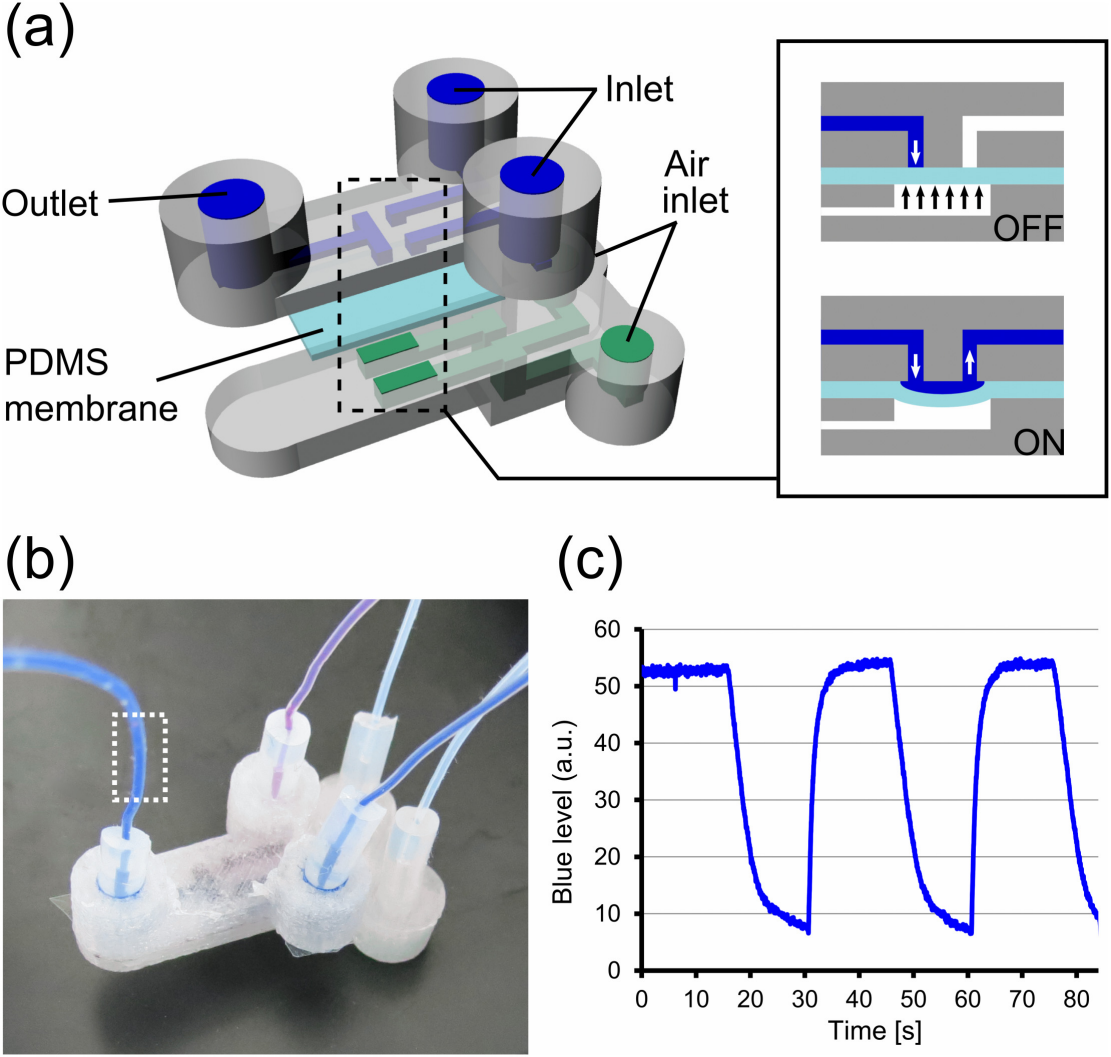
Flow selector device. (A) Schematic diagram of 3D printed flow selector device using PDMS membrane valve. (inset) Valves are closed when air pressure is applied from the bottom channel. When they are open, aqueous phases pumped at a constant pressure flow through the valve. (B) Fabricated flow selector device. (C) Plot of blue dye level measured at the outlet of the device, indicated as dotted square in (B). Deionized water with and without blue dye were switched every 15s.

Incorporation of active components into the millifluidic devices is crucial for the control of flow. Therefore we addressed this particular aspect by the incorporation of valve structures to both start and stop the flow of the aqueous phase within the 3D printed devices. Fig 3A and 3b show a schematic diagram and photograph of flow selector device that integrates valve structures. The valve itself was constructed by a polydimethylsiloxane (PDMS) membrane bonded to 3D printed parts. The PDMS membrane and 3D printed plastics were chemically bonded by treating the surface with oxygen plasma and (3-Aminopropyl)triethoxysilane (APTES) [28,29]. When the 3D printed parts were treated by oxygen plasma, areas around the valves were masked with black permanent marker pen to avoid bonding to the 3D printed parts. The masking can be removed by flushing ethanol in the fluidic channels.

The two liquids introduced from the inlets were switched by activating the corresponding valves exclusively. Liquids were pumped by pressure pumps at 180 mbar and valves were closed (i.e. “OFF” state) by applying pressure at 250 mbar to the membrane from underneath. To demonstrate the switching of flow, de-ionized water containing blue dye and pure water were introduced from the two inlets and the valves were operated. Color level, in particular blue content, of outgoing liquid was monitored at the tubing in the outlet (dotted square in Fig 3B). Fig 3C shows a time development of blue level as a result of the switching operation. It should be noted that the blue level quickly changed from the base level to near maximum. Approximately 85% of the maximum level was recovered within 1 s. In contrast, the blue level decreased rather slowly when switched from dye-containing water to pure water. This was due to a small (but concentrated) amount of dye remaining in the path between the valves and observation area, and this showed that the flow switching was happening instantly at the area next the valves.

### Modularized devices

**Fig 4:**
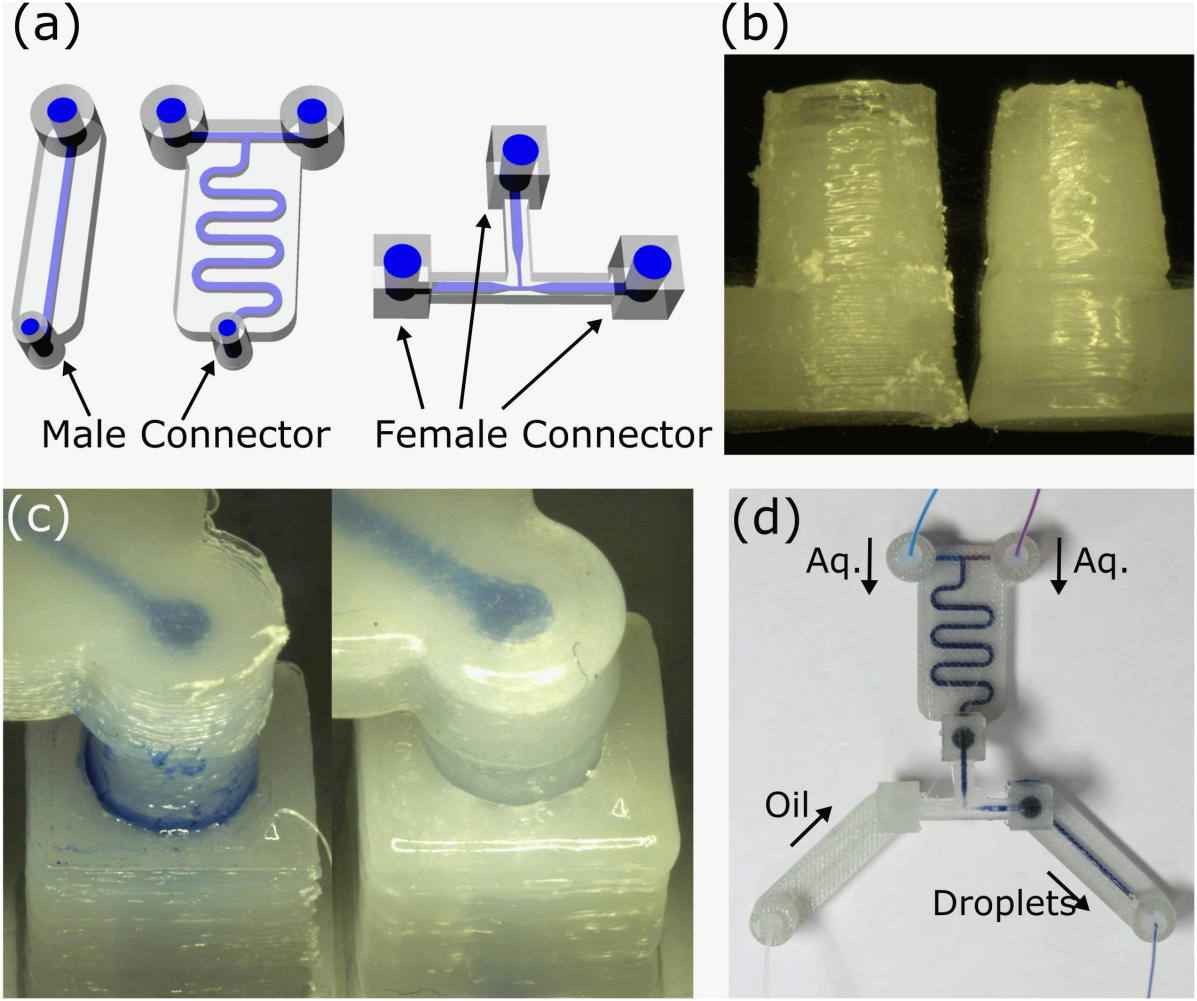
Interconnectable modular devices. (A) CAD designs of modularized 3D printed devices. (B) Surface roughness of 3D printed parts. Intact male connector (left) and vapor-treated connector (right). Both parts were first polished by a file. (C) Zoom-up of connected male/female parts. (left) intact parts (right) vapor-treated parts. (D) Assembled modular devices.

To exploit the potential versatility in design, manufacture and assembly we also set out to develop interconnecting parts that enable a ‘plug-and-play’ functionality for the 3D printed devices. To achieve this aim we developed modularized devices that designed and constructed by adding male and female connecting parts to the device design (Fig 4A). However, in reality, the 3D printed parts with such designs did not function properly due to the rough and hard surfaces of printed parts (Fig 4B). This surface roughness caused significant leakages between connected parts even after polished the surface by a file (Fig 4C left). Therefore, to create smooth and soft surfaces, we treated the 3D printed parts with dichloromethane (DCM) vapor for up to 15 min. A similar procedure using toluene was used to create smooth surfaces for machined microfluidics devices.[30] This process smoothens the surface of printed parts. Further, we found that this also created soft superficial layers. This meant that when vapor-treated male and female connectors are firmly put together, they are instantly well-sealed and formed a closed channel (Fig 4C right). For example, Fig 4D shows a picture of assembled modularized devices: a single input channel, dual input mixing channel, and droplet generator. The single channel module simply relays external input/output and male connector. The dual mixer takes two external inputs and mixes two liquids (e.g. red and blue-dye containing water). When assembled with a droplet generator with female connectors, they produced monodisperse W/O droplets encapsulating red and blue dyes. Additionally, PTFE tapes wrapped around male connectors helped to stop occasional leakages completely. Additionally, these male/female parts are designed as luer slip connections. Thus, non-3D printed components with industrial connections, such as plastic syringes, can be readily connected to these 3D printed parts without any leakages.

## Conclusions

In conclusion, we have demonstrated that 3D printed plastic devices can be used to carry out basic fluid manipulations and monitoring of biological experiments. Although the device is a proof-of-concept system, it should be emphasized that, to our best knowledge, this is the first ever demonstration of 3D printed fluidic devices using FDM-based 3D printers that produced W/O droplets for biological applications. Despite wide range of applications in microfluidic systems, W/O droplet generation was not achieved by other 3D printing methods due to the surface property of 3D printed materials. The use of hydrophilic FDM-based printing material made this possible. In addition, external components were integrated to the devices by exploiting the capability to construct 3D structure. In particular, we have shown that the integration of monitoring devices (i.e. sensors) and the construction of valve structures using chemical surface treatment were possible. It was also shown that the devices can be bonded together with soft PDMS membrane and networked via connecting parts to construct a larger, scalable devices. We think the technique to chemically bond 3D printed devices and PDMS is particularly important here because it makes possible to interface 3D printed millifluidic and PDMS-based microfluidic systems. While 3D printed devices enables easy fabrications of truly 3D structure, the printing resolution is relatively low. On the other hand, PDMS-based 2D microfluidic devices have better resolution, but it comes with laborious fabrication to construct 3D structures. As our method of these two devices can be easily achieved into any laboratories with typical microfluidics fabrication facility, we believe that our implementations here have paved the way to open up the immense potentials of 3D printed, integrated, and potentially larger-scale networked fluidic arrays.

## Materials and Methods

### Materials

Luria broth (LB) powder, dichloromethane, methylene blue and Sudan I were purchased from Sigma-Aldrich (UK) and used without further purification. FC40 was purchased from Flurochem (UK). PFPE-PEG-PFPE triblock co-polymer surfactant (EA surfactant) was a gift from Raindance Technologies (USA). Acrylonitrile butadiene styrene (ABS), Taulman 618 (nylon), high-impact polystyrene (HIPS) and polylactic acid (PLA) are purchased from Form Futura (Netherlands), polyethylene terephthalate (PET) was from Plastic2Print (Netherlands).

### Design and 3D Printing

Computer-aided design (CAD) models of devices incorporating various features were developed using Rhinoceros 5 (McNeel North America, USA). A digital stereolithographic and path planning rendering was created using Cura (Ultimaker, Netherlands). The Ultimaker Original and Ultimaker2 (Ultimaker, Netherlands) were used to construct 3D printed devices with a resolution of 0.1 mm per deposition layer.

For incorporation of a LED and photodiode (PD) sensor, a droplet incubator (Fig 2A) was first 3D printed with holes to accommodate LED and sensor. After printing, the holes was trimmed with files to smoothen the walls. A small amount of non-corrosive silicone adhesive (3145 RTV MIL-A-46146 sealant/adhesive, Dow Corning) was pasted on the cylindrical side of a LED and sensor and they were inserted into the holes of 3D printed devices. This step was carefully done so that the adhesive does not come in the optical pathway between LED and sensor and also has little contact with W/O droplets. The device with LED/sensor was left overnight to settle the adhesive to make the device leak-tight.

For modular device parts, 3D printed parts in PLA and PET were treated with dichloromethane (DCM) vapor for up to 15 min and 1 min, respectively, to create a smooth and soft superficial layer of the devices. DCM vapor was made in a large beaker on a hotplate set at 90°C and printed parts were kept in the beaker, but not directly in contact with liquid DCM.

All the CAD files designed here are available online [21].

### Preparation of cell culture

A day prior to experiment, *Escherichia coli* cell (MG1655 strain) was suspended from cryostock in liquid LB media and cultured at 37°C overnight. Cell suspension is diluted 100 fold prior to the experiment.

### Fabrication of 3D printed valve device

The device consists of 3D printed parts and a thin PDMS membrane (approx. 200–300 μm). The 3D printed parts are printed as described above. The PDMS membrane was made by mixing PDMS elastomer and curing agent at 10:1 ratio (by weight) and spincoated on a glass slide at 500rpm after degassed. It was based at 60°C for at least 2h.

Bonding of 3D printed parts and a PDMS membrane was done as follows: First, 3D printed parts are exposed to oxygen plasma (Harrick Plasma, USA) for 1 min and then immediately immersed in 5% (v/v) solution of (3-Aminopropyl)triethoxysilane (APTES) in water for 20 min at 50°C. After washed by water and completely dried under nitrogen stream, they were interfaced with a PDMS membrane, which was also exposed to oxygen plasma for 1 min. They were kept in close contact with a black clip for 1 h to bond.

Aqueous liquids (water containing blue and red dye) and air valves are actuated by pressure controller (MFCS-100, Fluigent, France). Aqueous phase was pumped at 180 millibar and air valves were at 250 millibar.

### Device Interfacing and Function

Extrinsic programmable syringe pumps (Aladdin-1000, World Precision Instruments, USA) with adjustable flow rates were interfaced with device inputs via 1 mm inner diameter polytetrafluoroethylene (PTFE; Cole-Parmer, USA) or silicone (Cole-Parmer, USA) tubes. Bacterial cells (*E*. *coli* strain MG1655) and liquid lysogeny broth (LB) culturing medium constituted the two aqueous phase inputs into the device. Liquid conveying channels throughout the devices had an inner diameter of 1 mm. Following aqueous phase diffusive mixing in winding channels, emulsion droplets formed at a T junction with immiscible oil phase. FC40 fluorinated oil with 2% v/v highly biocompatible tri-block perfluoropolyether-polyethyleneglycol-perfluoropolyether (PFPE-PEG-PFPE) copolymer EA surfactant was used to minimize coalescence of emulsion droplets at the phase interface. Sudan I red dye was used in place of cell media to visualize the aqueous phase.

### Data Acquisition and Analysis

For a droplet storage device (Fig 2A), a light emitting diode (LED; λ = 600nm) and photodiode (PD) couplet was integrated as part of an optical density (OD) sensing electronic circuit assembly. The LED and PD, positioned perpendicular to the droplet chamber. Current generated by the PD was converted to voltage and amplified by an op-amp circuit (Fig 2E). A programmable, computer-interfacing data acquisition (DAQ) device was used for the OD sensing electronics (NI USB-6009, National Instruments, UK). A calibration curve to convert voltage reading into OD_600_ was obtained using bacterial cultures with known ODs (S3 Fig). A custom-made LabVIEW software (National Instruments, UK) measured and save OD data in a PC.

Alternatively, OD measurement of cell growth in droplets was obtained at wavelength 600 nm using a NanoDrop 2000 (Thermo Scientific, USA) spectrophotometer, following dilution with the fluorinated oil and de-compartmentalization by centrifugation.

## Supporting Information

S1 Fig The minimum resolution of 3D Printed Devices. Actual dimensions of the 3D printed T-junction droplet generator. Printed in Ultimaker Original. Measured channel width were: (1) 1.09mm [1.2mm] (2) 0.40mm [0.4mm] (3) 0.24mm [0.4mm] (4) 0.39mm [0.4mm] (5) 1.12 mm [1.2mm] (values in square brackets indicate the width on the CAD software).
(TIF)

S2 Fig The reproducibility of 3D printed parts. The T-junction droplet generator (Fig 1A) was printed on Ultimaker 2 (N = 15) and variations in the width of sub-millimeter channels (positions indicated as (2), (3), and (4) in S1 Fig) were measured. The error bars were standard deviation. The numbers on the bars are coefficient of variables (C.V.).
(TIF)

S3 Fig Calibration curve for droplet cell growth device. The calibration curve (below) was obtained by measuring voltage readings of cell cultures. First, a serial 2-fold dilutions of fully-grown E.coli cell suspension in LB media was prepared (1x to 128x dilutions). OD600 of these dilutions were measured by a UV-Vis spectrophotometer. In the 3D printed monitoring device, a voltage reading of LB media from the attached photodiode (PD) sensor was adjusted to 3.5V by adjusting the supply voltage of LED attached in the device. Then, the most diluted (128x) cell culture was flushed into the device and a voltage from the PD sensor was measured. This process was repeated for the dilution series up to 1x culture (i.e., fully-grown cell suspension). After voltages were measured for each dilution, differences from pure LB media (i.e., 3.5V) were calculated and plotted with OD600 value.
(TIF)

S4 Fig An aerobic growth curve of E.coli in batch culture. E.coli cells were cultured in 1 L LB medium at 37°C in a shaking incubator. OD_600_ was measured by a UV-vis spectrophotometer.
(TIF)

## References

[1] Gibson I, Rosen DW, Stucker B. Additive manufacturing technologies. Springer; 2010.

[2] Paz VF, Emons M, Obata K, Ovsianikov A, Peterhänsel S, Frenner K, et al Development of functional sub-100 nm structures with 3D two-photon polymerization technique and optical methods for characterization. J Laser Appl. 2012;24: -. 10.2351/1.4712151

[3] Stampfl J, Liska R. New Materials for Rapid Prototyping Applications. Macromol Chem Phys. 2005;206: 1253–1256. 10.1002/macp.200500199

[4] Hanson Shepherd JN, Parker ST, Shepherd RF, Gillette MU, Lewis JA, Nuzzo RG. 3D Microperiodic Hydrogel Scaffolds for Robust Neuronal Cultures. Adv Funct Mater. 2011;21: 47–54. 10.1002/adfm.201001746 21709750 PMC3120232

[5] Ahn BY, Duoss EB, Motala MJ, Guo X, Park S-I, Xiong Y, et al Omnidirectional Printing of Flexible, Stretchable, and Spanning Silver Microelectrodes. Science (80-). 2009;323: 1590–1593. 10.1126/science.1168375 19213878

[6] Therriault D, White SR, Lewis JA. Chaotic mixing in three-dimensional microvascular networks fabricated by direct-write assembly. Nat Mater. 2003;2: 265–271. Available: 10.1038/nmat863 12690401

[7] Waldbaur A, Rapp H, Lange K, Rapp BE. Let there be chip-towards rapid prototyping of microfluidic devices: one-step manufacturing processes. Anal Methods. 2011;3: 2681–2716. 10.1039/C1AY05253E

[8] Symes MD, Kitson PJ, Yan J, Richmond CJ, Cooper GJT, Bowman RW, et al Integrated 3D-printed reactionware for chemical synthesis and analysis. Nat Chem. Nature Publishing Group; 2012; 1–6. 10.1038/nchem.1313 22522253

[9] Mathieson JS, Rosnes MH, Sans V, Kitson PJ, Cronin L. Continuous parallel ESI-MS analysis of reactions carried out in a bespoke 3D printed device. Beilstein J Nanotechnol. 2013;4: 285–291. 10.3762/bjnano.4.31 23766951 PMC3678396

[10] Kitson PJ, Symes MD, Dragone V, Cronin L. Combining 3D printing and liquid handling to produce user-friendly reactionware for chemical synthesis and purification. Chem Sci. 2013;4: 3099–3103. 10.1039/C3SC51253C

[11] Kitson PJ, Rosnes MH, Sans V, Dragone V, Cronin L. Configurable 3D-Printed millifluidic and microfluidic “lab on a chip” reactionware devices. Lab Chip. 2012; 10.1039/c2lc40761b 22875258

[12] Kitson PJ, Marshall RJ, Long D, Forgan RS, Cronin L. 3D Printed High-Throughput Hydrothermal Reactionware for Discovery, Optimization, and Scale-Up. Angew Chemie Int Ed. 2014; n/a–n/a. 10.1002/anie.201402654 25079230

[13] Chisholm G, Kitson PJ, Kirkaldy ND, Bloor LG, Cronin L. 3D printed flow plates for the electrolysis of water: an economic and adaptable approach to device manufacture. Energy Environ Sci. 2014; 10.1039/C4EE01426J

[14] Wijnen B, Hunt EJ, Anzalone GC, Pearce JM. Open-Source Syringe Pump Library. PLoS One. 2014;9: e107216 10.1371/journal.pone.0107216 25229451 PMC4167991

[15] Kolesky DB, Truby RL, Gladman AS, Busbee TA, Homan KA, Lewis JA. 3D bioprinting of vascularized, heterogeneous cell-laden tissue constructs. Adv Mater. 2014;26: 3124–30. 10.1002/adma.201305506 24550124

[16] Au AK, Bhattacharjee N, Horowitz LF, Chang TC, Folch A. 3D-printed microfluidic automation. Lab Chip. The Royal Society of Chemistry; 2015;15: 1934–41. 10.1039/c5lc00126a PMC4382387 25738695

[17] Lee KG, Park KJ, Seok S, Shin S, Kim DH, Park JY, et al 3D printed modules for integrated microfluidic devices. RSC Adv. The Royal Society of Chemistry; 2014;4: 32876 10.1039/C4RA05072J

[18] Gross BC, Erkal JL, Lockwood SY, Chen C, Spence DM. Evaluation of 3D printing and its potential impact on biotechnology and the chemical sciences. Anal Chem. American Chemical Society; 2014;86: 3240–53. 10.1021/ac403397r 24432804

[19] Anderson KB, Lockwood SY, Martin RS, Spence DM. A 3D printed fluidic device that enables integrated features. Anal Chem. American Chemical Society; 2013;85: 5622–6. 10.1021/ac4009594 23687961

[20] Erkal JL, Selimovic A, Gross BC, Lockwood SY, Walton EL, McNamara S, et al 3D printed microfluidic devices with integrated versatile and reusable electrodes. Lab Chip. The Royal Society of Chemistry; 2014;14: 2023–32. 10.1039/c4lc00171k PMC4436701 24763966

[21] Cronin Group. 3D Printed Reactionware Design Repository [Internet]. Available: http://www.chem.gla.ac.uk/cronin/media.php?media=react

[22] Agresti JJ, Antipov E, Abate AR, Ahn K, Rowat AC, Baret J-C, et al Ultrahigh-throughput screening in drop-based microfluidics for directed evolution. Proc Natl Acad Sci U S A. 2010;107: 4004–9. 10.1073/pnas.0910781107 20142500 PMC2840095

[23] Scanlon TC, Dostal SM, Griswold KE. A high-throughput screen for antibiotic drug discovery. Biotechnol Bioeng. 2014;111: 232–243. 10.1002/bit.25019 23955804 PMC4085149

[24] Baraban L, Bertholle F, Salverda MLM, Bremond N, Panizza P, Baudry J, et al Millifluidic droplet analyser for microbiology. Lab on a Chip. 2011 p. 4057 10.1039/c1lc20545e 22012599

[25] Chang D-E, Smalley DJ, Conway T. Gene expression profiling of Escherichia coli growth transitions: an expanded stringent response model. Mol Microbiol. 2002;45: 289–306. 10.1046/j.1365-2958.2002.03001.x 12123445

[26] Jakiela S, Kaminski TS, Cybulski O, Weibel DB, Garstecki P. Bacterial growth and adaptation in microdroplet chemostats. Angew Chemie—Int Ed. 2013;52: 8908–8911. 10.1002/anie.201301524 PMC3879160 23832572

[27] Sezonov G, Joseleau-Petit D, D’Ari R. Escherichia coli physiology in Luria-Bertani broth. J Bacteriol. 2007;189: 8746–9. 10.1128/JB.01368-07 17905994 PMC2168924

[28] Lee KS, Ram RJ. Plastic-PDMS bonding for high pressure hydrolytically stable active microfluidics. Lab Chip. 2009;9: 1618–1624. 10.1039/B820924C 19458871

[29] Ogilvie IRG, Sieben VJ, Cortese B, Mowlem MC, Morgan H. Chemically resistant microfluidic valves from Viton[registered sign] membranes bonded to COC and PMMA. Lab Chip. 2011;11: 2455–2459. 10.1039/C1LC20069K 21617822

[30] Ogilvie IRG, Sieben VJ, Floquet CFA, Zmijan R, Mowlem MC, Morgan H. Reduction of surface roughness for optical quality microfluidic devices in PMMA and COC. J Micromechanics Microengineering. 2010;20: 65016 Available: http://stacks.iop.org/0960-1317/20/i=6/a=065016

