# Supplementary figures and images for: Customizable 3D Printed ‘Plug and Play’ Millifluidic Devices for Programmable Fluidics
Source: PLoS One. 2015 Nov 11;10(11):e0141640. doi: 10.1371/journal.pone.0141640 (PMC4641590; doi:10.1371/journal.pone.0141640)

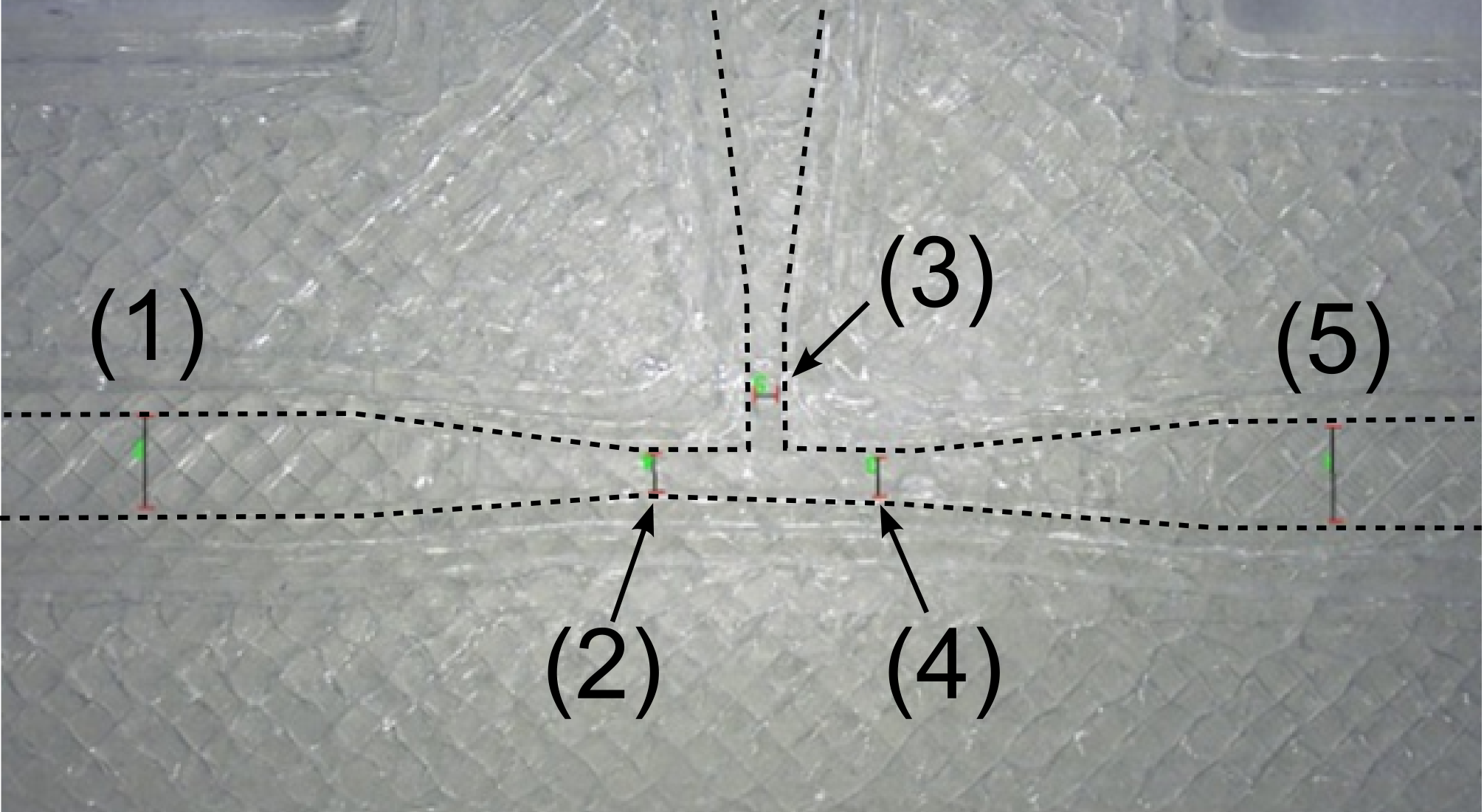

Supplement: S1 Fig — Actual dimensions of the 3D printed T-junction droplet generator. Printed in Ultimaker Original. Measured channel width were: (1) 1.09mm [1.2mm] (2) 0.40mm [0.4mm] (3) 0.24mm [0.4mm] (4) 0.39mm [0.4mm] (5) 1.12 mm [1.2mm] (values in square brackets indicate the width on the CAD software). (TIF) [file pone.0141640.s001.tif]

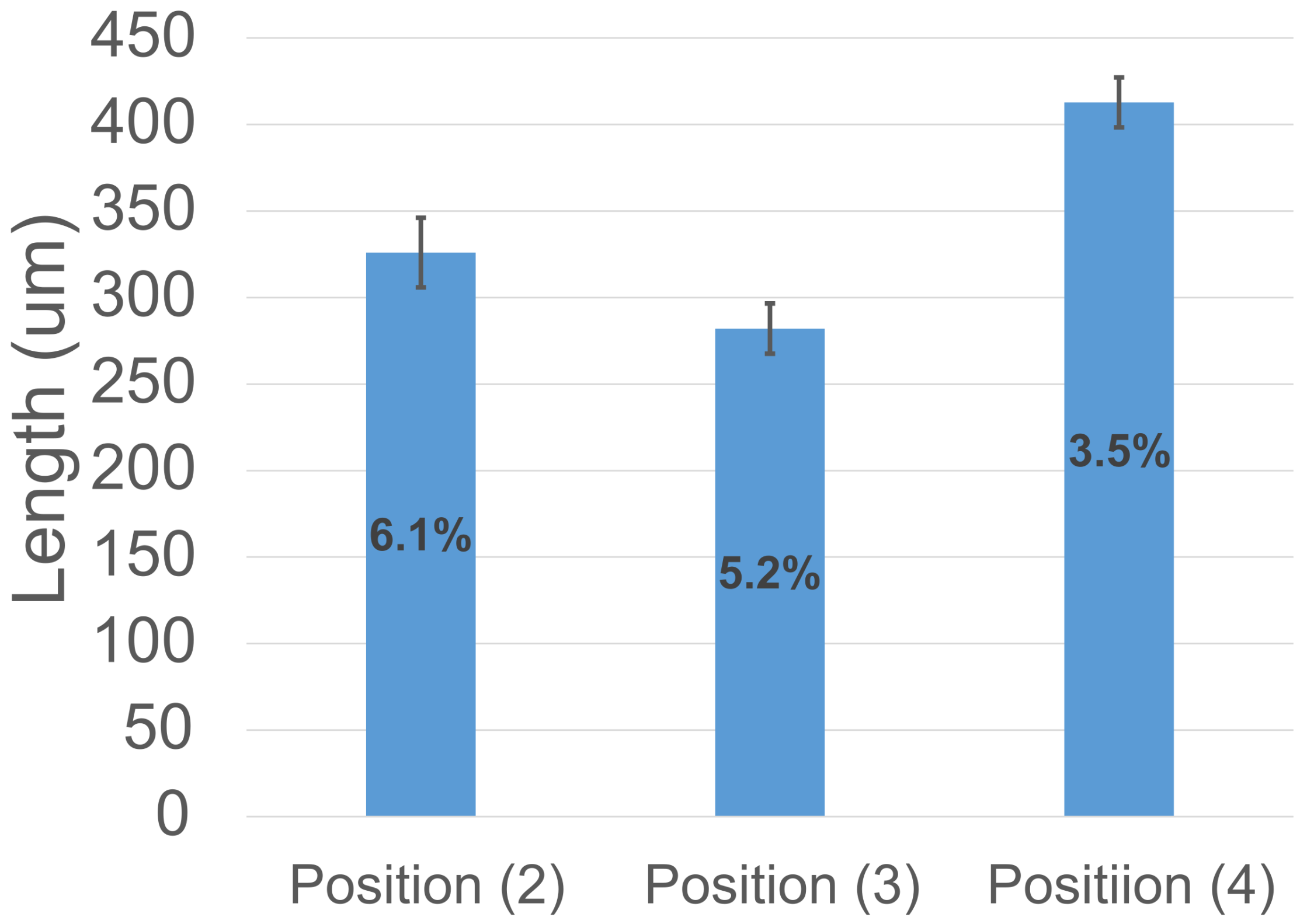

Supplement: S2 Fig — The T-junction droplet generator (Fig 1A) was printed on Ultimaker 2 (N = 15) and variations in the width of sub-millimeter channels (positions indicated as (2), (3), and (4) in S1 Fig) were measured. The error bars were standard deviation. The numbers on the bars are coefficient of variables (C.V.). (TIF) [file pone.0141640.s002.tif]

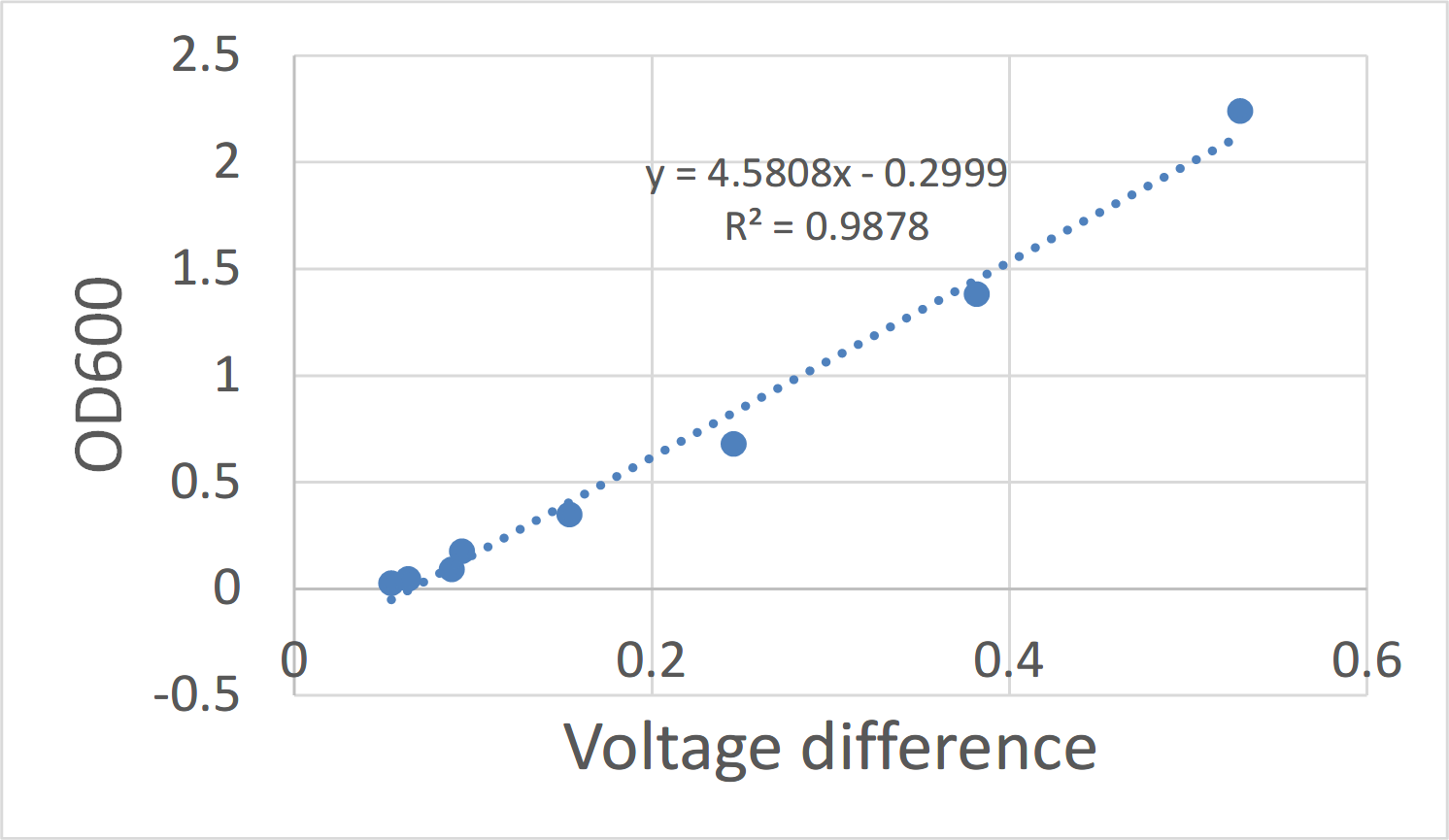

Supplement: S3 Fig — The calibration curve (below) was obtained by measuring voltage readings of cell cultures. First, a serial 2-fold dilutions of fully-grown E.coli cell suspension in LB media was prepared (1x to 128x dilutions). OD600 of these dilutions were measured by a UV-Vis spectrophotometer. In the 3D printed monitoring device, a voltage reading of LB media from the attached photodiode (PD) sensor was adjusted to 3.5V by adjusting the supply voltage of LED attached in the device. Then, the most diluted (128x) cell culture was flushed into the device and a voltage from the PD sensor was measured. This process was repeated for the dilution series up to 1x culture (i.e., fully-grown cell suspension). After voltages were measured for each dilution, differences from pure LB media (i.e., 3.5V) were calculated and plotted with OD600 value. (TIF) [file pone.0141640.s003.tif]

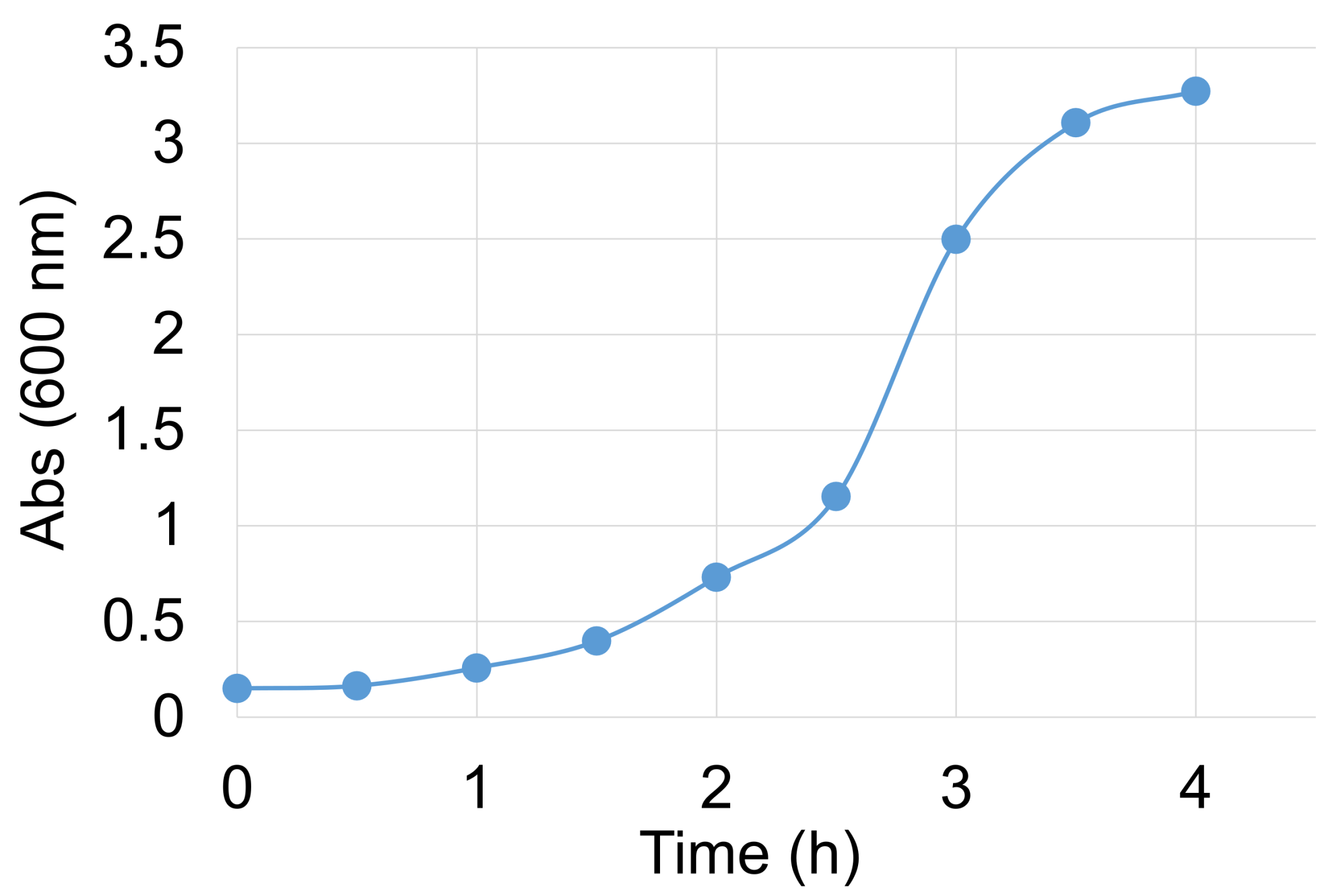

Supplement: S4 Fig — E.coli cells were cultured in 1 L LB medium at 37°C in a shaking incubator. OD600 was measured by a UV-vis spectrophotometer. (TIF) [file pone.0141640.s004.tif]
